# Safety and quality evaluation of commercial wheat flour in Saudi Arabia through microbiological, chemical, and molecular analyses: a preliminary baseline assessment

**DOI:** 10.3389/fmicb.2026.1930002

**Published:** 2026-08-31

**Authors:** Abdullah A. Khan Ghyathuddin, Faisal Al-Sarraj, Atif Bamagoos, Nada M. Nass, Ehab Mattar, Abdullah Baz, Ibrahim Alotibi, Ali Shaibah, Mohammed A. Almatry, Saleh M. Al-Maaqar

**Affiliations:** 1 Department of Biological Sciences, Faculty of Science, King Abdulaziz University, Jeddah, Saudi Arabia; 2 Immunology Unit, King Fahd Medical Research Centre, King Abdulaziz University, Jeddah, Saudi Arabia; 3 Department of Health Information Technology, Applied College, King Abdulaziz University, Jeddah, Saudi Arabia; 4 Department of Animal Production, Faculty of Agriculture, Sana’a University, Sana’a, Yemen; 5 Department of Biology, Faculty of Education, Albaydha University, Al-Baydha, Yemen

**Keywords:** 16S rRNA, heavy metals, hemolytic activity, microbial quality, mycotoxins, pesticide residues, Saudi Arabia, wheat flour

## Abstract

Wheat flour is a global staple food commodity, yet its microbiological and chemical safety remains a public health concern due to potential contamination throughout the production and supply chain. This study provides a preliminary baseline assessment of the quality and safety of 14 commercial wheat flour samples (X1–X14) obtained from markets in Jeddah, Saudi Arabia. Standard microbiological methods were employed to detect pathogenic indicators. Heavy metal concentrations were quantified. Ochratoxin-A and total aflatoxins (B1 + B2 + G1 + G2) were analyzed, while pesticide residues were determined. A total of 23 bacterial isolates were purified and identified via 16S rRNA gene Sanger sequencing, with phylogenetic trees constructed using MEGA12, and hemolytic activity was assessed on sheep blood agar. All samples were negative for *Salmonella* spp., *Staphylococcus aureus* (*S. aureus*), *Bacillus cereus* (*B. cereus*), *and Escherichia coli* (*E. coli*) were below 10 CFU/g, indicating compliance with international safety standards. However, yeast and mold counts varied significantly, with one sample (X8) exhibiting a critically high count (830 CFU/g). Heavy metal levels were generally within Codex Alimentarius limits, though one sample contained elevated lead (0.566 mg/kg). Ochratoxin-A was detected in Three samples (X1: 0.17 μg/kg; X2: 0.12 μg/kg; X9: 0.14 μg/kg), while total aflatoxins were absent from all samples. The most prevalent pesticide residue was deltamethrin (X2, X3, X4, X6; range 0.015–0.059 mg/kg), followed by chlorpyrifos ethyl (X3: 0.009 mg/kg; X6: 0.008 mg/kg). Molecular identification revealed a taxonomically diverse bacterial community dominated by the genus *Staphylococcus* (*13/23 isolates*, *56.5%*), *followed by Rothia kristinae* (*3/23*), *Bacillus paramycoides* (*2/23*), *Peribacillus frigoritolerans* (*1/23*), *and other genera* (*4/23*). Hemolytic activity testing revealed that all staphylococcal isolates were gamma-hemolytic (non-hemolytic), whereas the three *Bacillus* isolates (*Bacillus paramycoides* and *Peribacillus frigoritolerans*) exhibited beta-hemolysis. These preliminary findings underscore the necessity for routine monitoring of post-harvest handling, storage conditions, and pesticide residues to ensure consumer safety. Due to the limited sample size (*n* = 14), future studies with broader geographic and seasonal coverage are recommended.

## Graphical abstract

**Figure:**
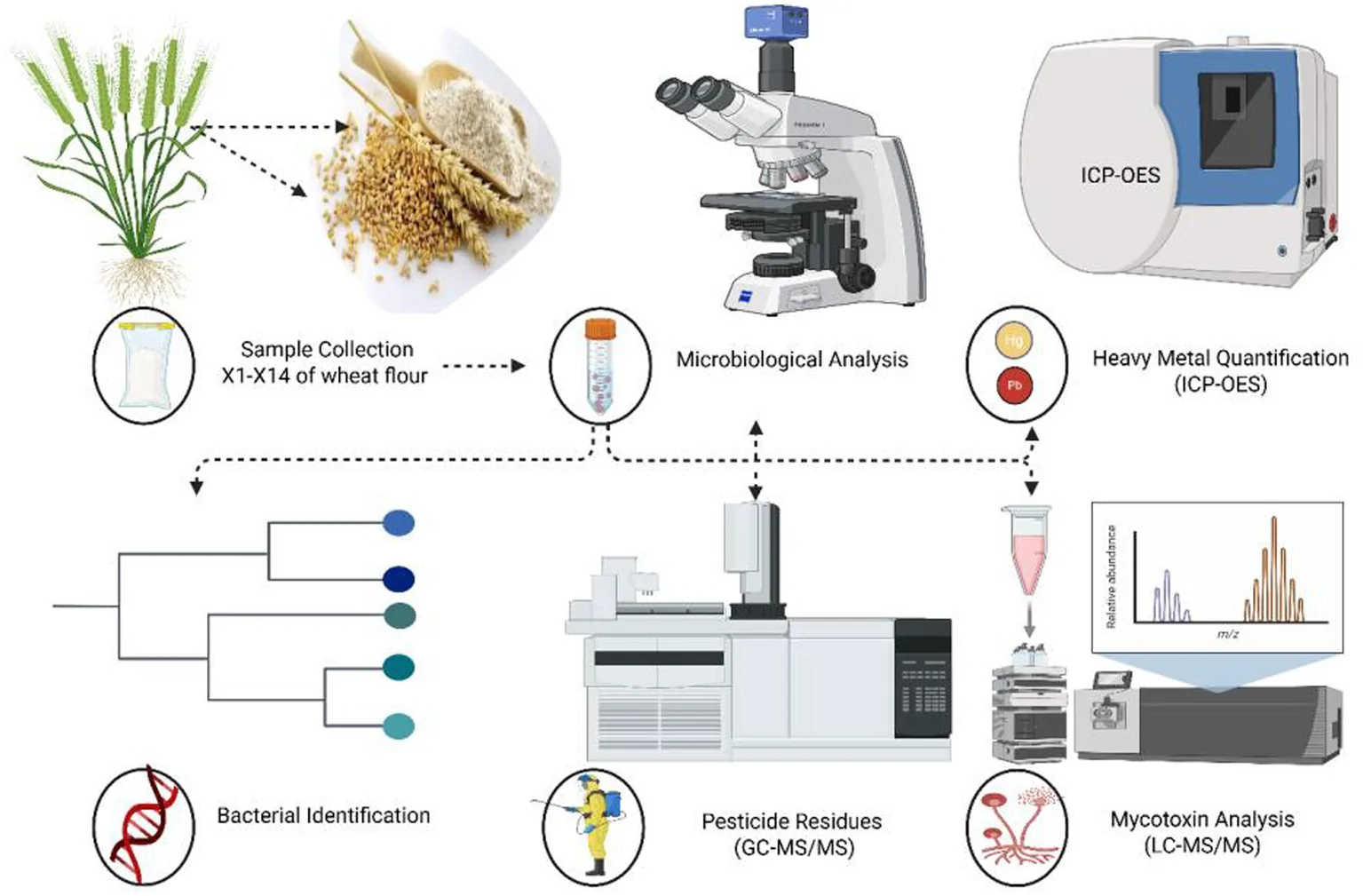

## 1 Introduction

Wheat (*Triticum aestivum* L.) is also one of the most commonly grown cereal crops in the world, as it is a staple food of billions of people. Wheat flour is utilized in bread, pastries, noodles, infant formulas, and many other food items of around 800 million metric tons annually (Ahmadi et al., 2025). Although wheat flour has a significant nutritional and economic value, it can be contaminated by microbes and chemicals during various parts of the processing process, such as harvesting, milling, packaging, and storage (Rivera et al., 2025).

Recent studies have pointed out that wheat foods have become a major source of food poisoning. A thorough examination by Rivera et al. (2025) revealed that Shiga toxin-producing *Escherichia coli* (STEC) and Salmonella could survive in wheat flour over long periods with survival duration up to two years because of low water activity of the flour (usually 0.491–0.619). The outbreak of foodborne illnesses related to contaminated wheat flour has been reported more frequently in the global context, such as an outbreak of *E. coli* O157: H7 in raw cookie dough in the United States (2016) and a *Salmonella Agbeni* outbreak in cake mix (2018) (Magallanes Lopez and Simsek, 2021). Australian, European, and North American research has repeatedly detected *Salmonella* spp., *E. coli*, *B. cereus*, and other microorganisms in flour, causing spoilage (Berghofer et al., 2003; Eglezos, 2010; Zhang et al., 2021).

In addition to microbiological hazards, heavy metal content of wheat flour is also a major threat to human health. The heavy metals such as lead (Pb), cadmium (Cd), and arsenic (As) are contaminated to the soil, water used in irrigation, atmospheric deposition, and agricultural practices (Mustfa and Ahmed Albarwary, 2025). Recent research by Xing et al. (2025) has shown that the consumption of fermented Cd- and Pb-contaminated wheat flour poses a significant health risk, with fermentation having a two-fold effect on the relative bioavailability of Cd. This observation is especially pertinent because fermentation is popular in the baking of bread, pita, naan, and other traditional baked goods. Moreover, a process-based investigation conducted by Kayisoglu et al. (2025) monitored heavy metal redistribution during wheat milling and discovered that some metals (Cr and Cu) were produced in the crushing phases, presumably due to equipment abrasiveness, highlighting the need to keep equipment in good condition.

A new issue in food safety is that raw wheat flour could be a reservoir of antibiotic resistance genes. Gargano et al. (2024) examined the transmission of antimicrobial resistance genes in raw materials to sourdough breads and discovered that some of the semolina samples and the respective doughs had tetracycline resistance genes *tet*(A) and *tet*(B). It means that raw wheat milling products may contain resistance determinants and the horizontal gene transfer to pathogenic bacteria may occur.

Food safety authorities in the Gulf region have realized the need to develop holistic standards of wheat flour. Recently, a technical regulation of wheat flour that is to be consumed directly by people has been developed by the Saudi Food and Drug Authority in collaboration with other states of the Gulf Cooperation Council as a sign of increased awareness of flour safety as a health concern of the population. Even with these changes, there is still a paucity of information on the microbiological quality, molecular characterization of microbial communities, heavy metal contamination levels, and virulence potential of flour-related bacteria in the Saudi market. Thus, the purpose of the present research was to provide a preliminary assessment of the quality and safety of commercial wheat flour available in Jeddah, Saudi Arabia, by performing microbiological, quantitative determination of heavy metals, molecular, and hemolytic activity.

## 2 Materials and methods

### 2.1 Sample collection

A total of fourteen commercial wheat flour samples were obtained from local retail markets in Jeddah, Saudi Arabia, between May and November 2025. Samples were collected using a convenience sampling approach from major retail supermarkets. All samples were purchased in their original, unopened packaging from retail shelves. Immediately after purchase, samples were transported to the laboratory in insulated coolers at ambient temperature and stored at 22 °C–25 °C (room temperature) in a dry, dark environment until analysis, as recommended by the testing laboratory for flour samples. All analyses were initiated within 24 h of sample receipt at the testing laboratory. Samples from the same batch were not available; each sample represents a different commercially available product. The sample set included both locally produced (*n* = 10) and imported (*n* = 4) brands, representing various types of flour: brown flour, whole wheat flour, fermented flour, premium flour, super-premium flour, pastry flour, and patent flour. The imported samples originated from the United Arab Emirates (*n* = 2), Turkey (*n* = 1), and Egypt (*n* = 1), as indicated on product packaging. Detailed sample information, including brand names, net weight, flour types, fortification status, and batch numbers, is shown in Table 1.

**Table 1:** Description and source information for local and imported wheat flour samples.

Sample code	Flour ID	Sources	Flour type (English)	Fortification	Batch No.	Net weight
X1	F1	Local	Brown Flour (All Purpose)	Iron, Vitamins	6,287,017,150,414	1 kg
X2	F2		Premium Flour (All Uses)	Not specified	6,287,017,150,599	1 kg
X3	F3		Wheat flour	Iron, Vitamins	6,285,021,000,251	1 kg
X4	F4		Super Premium (All Uses)	Not specified	6,287,017,150,032	1 kg
X5	F5		Flour (All Purpose)	Not specified	6,287,019,400,180	1 kg
X6	F6		Flour (All Purpose)	Not specified	6,291,003,058,103	1 kg
X7	F7		Premium Flour (All Uses)	Iron, Vitamins	6,287,017,150,377	1 kg
X8	F8		Brown Flour (Multi-Purpose)	Not specified	6,287,019,400,272	1 kg
X9	F9		Fermented flour (All Purpose)	Not specified	6,281,100,610,168	1 kg
X10	F10		Superior Flour (All Purpose)	Iron, Vitamins B1, B2, B3, B9, D	6,287,019,070,154	1 kg
X11	F11	Imported	Superior Flour (All Purposes)	Not specified	6,287,019,400,234	1 kg
X12	F12		For All Pastry Needs	Not specified	6,281,014,960,038	1 kg
X13	F13		To bake all kinds of luxury desserts	Not specified	6,281,014,960,014	1 kg
X14	F14		Patent Flour (All Purposes)	Iron, Vitamins B1, B2, B3, B9, D	6,287,019,070,345	1 kg

### 2.2 Microbiological analysis

Microbiological analyses were conducted at Laboratory KSA, an ISO/IEC 17025 accredited third-party laboratory, following standard methods. Sample preparation: 25 g of each flour sample was aseptically weighed and mixed with 225 mL of Buffered Peptone Water (BPW) in a sterile stomacher bag. The mixture was homogenized for 2 min using a Stomacher® 400 circulator (Seward, UK). For the enumeration of *Bacillus cereus*, *E. coli,* and *S. aureus*, serial ten-fold dilutions (up to 10^−3^) were prepared in 0.1% peptone water. *Salmonella* detection was performed according to ISO 6579-1:2017. Samples were serially diluted, and 1 mL aliquots were mixed with molten VRBA (45 °C–50 °C) in sterile Petri dishes. After solidification, plates were incubated at 37 °C for 24 h. Dark red colonies with or without bile precipitate were counted as presumptive *E. coli*. *S. aureus* was enumerated according to ISO 6888-1:1999 (Amendment 2:2018). Yeast and mold counts were determined according to ISO 21527-1:2008. All microbiological analyses were performed in duplicate.

### 2.3 Heavy metal analysis

Heavy metal concentrations (cadmium, lead, arsenic, mercury) were quantified using Inductively Coupled Plasma Optical Emission Spectrometry (ICP-OES) (Agilent 5,100, Agilent Technologies, Santa Clara, CA, USA). Sample digestion was performed using a microwave digestion system (Mars 6, CEM Corporation, Matthews, NC, USA) with concentrated nitric acid (65%, Suprapur® grade) and hydrogen peroxide (30%). The following wavelengths were used for quantification: Pb 220.353 nm, Cd 228.802 nm, As 188.980 nm, and Hg 253.652 nm. Plasma conditions were RF power 1.2 kW, plasma flow 15 L/min, auxiliary flow 1.5 L/min, and nebulizer flow 0.7 L/min.

Quality control: Calibration curves were prepared using certified reference standards (1,000 mg/L single-element standards, Merck KGaA, Darmstadt, Germany). Certified Reference Material (CRM) NIST SRM 1567b Wheat Flour was analyzed with each digestion batch to validate accuracy. Recovery rates ranged from 92–106% for all four metals. The limits of detection (LOD) and quantification (LOQ) were determined based on 3 and 10 times the standard deviation of the blank, respectively: Pb (LOD 0.003, LOQ 0.010 mg/kg), Cd (LOD 0.002, LOQ 0.006 mg/kg), As (LOD 0.005, LOQ 0.015 mg/kg), and Hg (LOD 0.004, LOQ 0.012 mg/kg). The reported results (e.g., <0.01 mg/kg) represent concentrations below the LOQ but above the LOD. Method precision was assessed by analyzing a quality control sample in replicate (*n* = 5), with relative standard deviation (RSD) of <5% for all metals. All heavy metal analyses were performed in duplicate.

### 2.4 Mycotoxin and pesticide residue analysis

Mycotoxin and pesticide residue analyses were performed at Laboratory KSA using validated methods within the laboratory’s accredited scope. The mycotoxin testing panel included aflatoxin B1 (AFB1), aflatoxin B2 (AFB2), aflatoxin G1 (AFG1), aflatoxin G2 (AFG2), and ochratoxin A (OTA), which represented the mycotoxins routinely analyzed in wheat flour under the laboratory’s accredited scope and for which certified reference materials were available. Quantification of OTA and the individual aflatoxins AFB1, AFB2, AFG1, and AFG2 was performed using liquid chromatography–tandem mass spectrometry (LC–MS/MS) on a Shimadzu LCMS-8050 system (Shimadzu Corporation, Kyoto, Japan). Total aflatoxins were calculated as the sum of AFB1, AFB2, AFG1, and AFG2. Flour samples were extracted using an acetonitrile/water mixture %60/30, followed by immunoaffinity column clean-up. Chromatographic separation was achieved using a Phenomenex Kinetex C18 column (100 × 2.1 mm, 2.6 μm) maintained at 40 °*C. mobile* phase A consisted of water containing 0.1% formic acid, whereas mobile phase B consisted of methanol containing 0.1% formic acid. The initial mobile-phase composition was 80:20 (A:B). The gradient program was 20% B from 0 to 2 min, followed by a linear increase from 20% to 100% B from 2 to 10 min, maintenance at 100% B from 10 to 12 min, and return to the initial 20% B condition from 12 to 15 min. The flow rate was 0.3 mL/min, the injection volume was 10 μL, and the total chromatographic run time was 15 min. Detection was performed using electrospray ionization (ESI) in positive-ion mode with multiple reaction monitoring (MRM) of analyte-specific transitions. The laboratory-reported limits of quantification were 0.1 μg/kg for OTA and 0.5 μg/kg for total aflatoxins.

Pesticide residues, including deltamethrin, bifenthrin, and chlorpyrifos ethyl, were determined using multi-residue analysis. Sample preparation was performed using the QuEChERS (Quick, Easy, Cheap, Effective, Rugged, and Safe) procedure based on AOAC Official Method 2007.01, followed by gas chromatography–tandem mass spectrometry (GC–MS/MS) using an Agilent 7890B gas chromatograph coupled to an Agilent 7000D triple-quadrupole mass spectrometer (Agilent Technologies).

Chromatographic separation was performed using an Agilent J&W DB-5MS column (30 m × 0.25 mm i.d., 0.25 μm film thickness). Helium was used as the carrier gas at a constant flow rate of 1.2 mL/min. The oven temperature was initially maintained at 60 °C for 1 min, increased to 170 °C at 40 °C/min, and subsequently increased to 310 °C at 5 °C/min, with a final hold of 5 min. A 1 μL aliquot was injected in splitless mode. Mass-spectrometric detection was performed using electron-impact ionization at 70 eV and MRM, with two analyte-specific transitions monitored for each pesticide the method detection limits for pesticide residues ranged from 0.003 to 0.005 mg/kg, depending on the compound. Mycotoxin concentrations were expressed in μg/kg, whereas pesticide residue concentrations were expressed in mg/kg.

### 2.5 Bacterial isolation and purification

**Figure 1:**
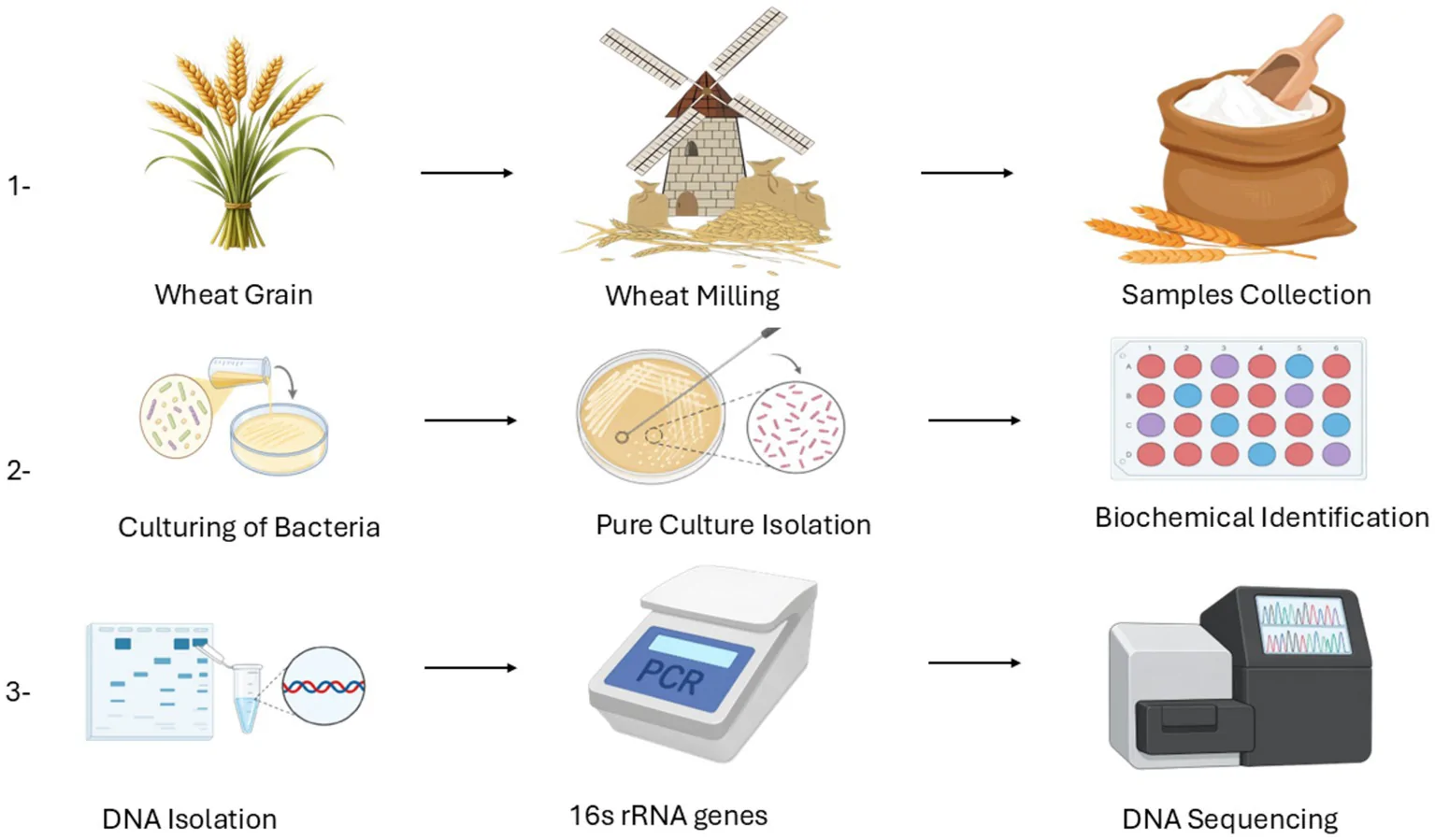
Workflow diagram of bacterial isolation, purification, and molecular identification from commercial wheat flour samples.

Figure 1 illustrates the bacterial isolation and purification workflow, followed by conventional phenotypic characterization and molecular identification via 16S rRNA gene sequencing. From each flour sample, manual serial dilutions were plated onto Nutrient Agar (NA) and Tryptic Soy Agar (TSA) and incubated at 37 °C for 24–48 h. Morphologically distinct colonies were selected and sub-cultured onto fresh agar plates to obtain pure isolates. A total of 23 pure bacterial isolates were selected for further characterization and designated A1 through A23. Stock cultures were maintained at −80 °C in 20% glycerol for long-term preservation.

### 2.6 Phenotypic characterization

Gram staining was performed using standard protocols. Cell shape, arrangement, and Gram reaction were recorded under oil immersion microscopy (1,000× magnification). Colony characteristics were described following 24–48 h of growth on TSA at 37 °C. A loopful of fresh bacterial culture was mixed with 3% hydrogen peroxide on a clean glass slide. Immediate bubble formation indicated a positive catalase reaction. Oxidase activity was determined by oxidase reagent on filter paper. Development of a purple color within 10–30 s indicated a positive reaction. Hemolytic activity was assessed on sheep blood agar (5% defibrinated sheep blood). Isolates were streaked onto blood agar and incubated at 37 °C for 24–48 h. Hemolysis was classified based on the appearance of the zones around the colonies: *α*-hemolysis (partial clearing with a greenish discoloration), *β*-hemolysis (complete, clear lysis of red blood cells, resulting in a transparent zone), and *γ*-hemolysis (no visible zone of clearing). All assays were performed in duplicate.

### 2.7 Molecular identification (16S rRNA gene sequencing)

#### 2.7.1 Genomic DNA extraction

Bacterial cultures were grown in LB broth at 37 °C with shaking (200 rpm) until the optical density at 600 nm (OD600) reached 0.8–1.0 (mid-log phase). Extraction of DNAs was carried out by 10 mL of each isolated strain. Following incubation, 10 mL of each bacterial culture was transferred to a Falcon tube and centrifuged at 7,500 rpm for 10 min to pellet the cells. The supernatants were carefully discarded, and the cell pellets were resuspended in 300 μL of lysis buffer (TES) consisting of 100 mM Tris base (pH 8), 50 mM EDTA, and 10% SDS. The resuspended cells were then transferred to 1.5 mL microcentrifuge tubes. Subsequently, 20 μL of lysozyme (20 mg/mL) was added to each tube, and the mixture was incubated at 37 °C for 2 h. Following this, 20 μL of proteinase K (20 mg/mL) was added, and the tubes were incubated at 37 °C for an additional 2 h. To the lysed cells, 300 μL of sodium acetate 3 M was added, gently mixed, and then incubated at −20 °C for 10 min. Following this step, 500 μL of chloroform: isoamyl alcohol mixture (24:1 ratio) was added to each tube, and the solutions were mixed gently for 10 min by inversion. The mixture was then centrifuged at 12,000 rpm for 15 min to separate the phases. The upper aqueous phase containing the DNA was carefully transferred to a sterile 1.5 mL microcentrifuge tube, ensuring not to disturb the interface layer. To precipitate the DNA, an equal volume of isopropanol (pre-cooled to −20 °C) was added to each tube and mixed gently by inversion. The tubes were then incubated at −80 °C for 1 h to facilitate DNA precipitation. After incubation, the tubes were centrifuged at 12,000 rpm for 15 min to pellet the DNA. The supernatants were discarded, and the DNA pellets were washed with 70% ethanol to remove residual salts. Care was taken to decant the ethanol thoroughly, and the DNA pellets were air-dried at room temperature or in a 50 °C incubator. It is crucial not to over-dry the pellets, as this may hinder their dissolution in the subsequent step. Finally, the DNA pellets were resuspended in 50 μL of Tris-EDTA (TE) buffer and allowed to sit overnight at 4 °C to ensure complete dissolution. The evaluation of DNA purity via A260/A280 ratio using NanoDrop 7,000 Spectrophotometer (Thermo Fisher Scientific, Waltham, MA, USA), and DNA safety was checked by 1% agarose gel electrophoresis.

#### 2.7.2 Sequence and phylogenetic analysis

The 16S rRNA gene was amplified using a thermal cycler (Applied Biosystems Veriti™ 96-Well Thermal Cycler, Thermo Fisher Scientific) by using 16S rRNA universal forward and reverse primers, 27F (AGAGTTTGATCMTGGCTCAG) and 1492R (TACGGYTACCTTGTTACGACTT). Fifty μl of reaction mixture contining (1 μL DNA templet, 1 μL primers, 25 μL green PCR mix Promega, Go Taq R Green Master Mix, USA), and 22 μL RNase-free water. PCR conditions consisted of an initial denaturation at 95 °C for 5 min, followed by 36 cycles of denaturation (94 °C for 40 s), annealing (58 °C for 40 s), and extension (72 °C for 90 Sec), and a final extension at 72 °C for 10 min. The amplification product was examined by 1% agarose gel electrophoresis, and the gel was visualized by Ultra-Violet Product (UVP Bio Spectrum ® Imaging System) (Yanez et al., 2003; Momtaz and Yadollahi, 2013). Sequencing of the PCR amplicons of the isolates was done by Macrogen, Inc. (Seoul, Korea) by Sanger sequencing. The 16S rRNA sequence data were compared with available sequence data in the GenBank at the National Center of Biotechnology Information (NCBI) using the Basic Local Alignment Search Tool (BLAST) program. The isolates were identified based on the sequence similarity to their closest homolog (Altschul et al., 1990). MEGA12 software used for phylogenetic analysis and align MUSCLE for multiple sequence alignment, then the tree topology validated using NJ method with 1,000 bootstrap for constructing the phylogenetic tree (Kumar et al., 2024). Bootstrap values % were considered well-supported; lower values were interpreted with caution.

### 2.8 Statistical analysis

All microbiological and chemical analyses were performed in duplicate. Results are expressed as mean values. Descriptive statistics (mean, SD, median, range) were calculated for all parameters. For contaminants detected above the LOD, mean concentrations were compared against SFDA/GSO and Codex limits using one-sample t-tests. Microbiological counts below the LOR (<10 CFU/g) were treated as 5 CFU/g. A *p*-value < 0.05 was considered significant. Phylogenetic trees were constructed using MEGA12 with 1,000 bootstrap replicates. Bootstrap values ≥70% were well-supported; lower values were interpreted with caution.

## 3 Results

### 3.1 Microbiological quality of flour samples

The findings of fourteen commercial wheat flour samples were microbiologically analyzed, and the results showed that all of the samples met food safety regulations with regard to harmful microorganisms (Table 2). None of the 25 g samples tested contained any *Salmonella* spp. All samples had levels of *Bacillus cereus*, *Escherichia coli*, and *Staphylococcus aureus* below the Limit of Reporting (LOR = 10 CFU/g), suggesting that these pathogens were either absent or extremely low. However, there were significant differences in the amounts of mold and yeast between samples (Table 2). Notably, Flour X8 significantly exceeded permissible limits for wheat flour with extremely high yeast and mold counts (830 CFU/g). Elevated amounts were seen in Flour X9 (100 CFU/g). Flour X7 displayed 70 CFU/g. Flour X4 displayed 60 CFU/g. Flours X1 and X2 showed moderate counts (50 CFU/g each). X3, X6, and X10 showed low levels (<10 CFU/g to 10 CFU/g).

**Table 2:** Microbiological investigation results of wheat flour samples.

Sample ID	*Bacillus cereus* (CFU/g)	*E. coli* (CFU/g)	*Salmonella* spp. (in 25 g)	*S. aureus* (CFU/g)	Yeast & molds (CFU/g)
X1	<10	<10	Not detected	<10	50
X2	<10	<10	Not detected	<10	50
X3	<10	<10	Not detected	<10	<10
X4	<10	<10	Not detected	<10	60
X5	<10	<10	Not detected	<10	30
X6	<10	<10	Not detected	<10	<10
X7	<10	<10	Not detected	<10	70
X8	<10	<10	Not Detected	<10	830
X9	<10	<10	Not detected	<10	100
X10	<10	<10	Not detected	<10	10
X11	<10	<10	Not detected	<10	50
X12	<10	<10	Not detected	<10	60
X13	<10	<10	Not detected	<10	70
X14	<10	<10	Not detected	<10	40

### 3.2 Heavy metal analysis

Descriptive statistics for heavy metal concentrations. Lead levels varied considerably across samples, with a mean of 0.128 ± 0.158 mg/kg (median 0.066 mg/kg; range <0.01–0.566 mg/kg). Grubbs’ test identified sample X1 (Brown Flour, 0.566 mg/kg) as a statistical outlier (G = 3.42, *p* < 0.05). After exclusion of this outlier, the mean lead concentration was 0.065 ± 0.052 mg/kg, which was significantly below the GSO limit of 0.20 mg/kg (one-sample *t*-test, *t* = 2.50, *df* = 12, *p* = 0.030). Cadmium levels (0.033 ± 0.014 mg/kg; range 0.01–0.05 mg/kg) were also significantly below the GSO limit of 0.10 mg/kg (one-sample *t*-test, *t* = −18.4, *df* = 13, *p* < 0.001). Arsenic was detected only in samples X7 and X10 (0.01 mg/kg each), with all other samples <0.01 mg/kg. Mercury was detected only in sample X7 (0.03 mg/kg), with all other samples <0.01 mg/kg.

### 3.3 Mycotoxin and pesticide residue contamination

Mycotoxin and pesticide residue analysis revealed variable contamination levels across the 14 samples (Table 3). Ochratoxin-A was detected in three samples: X1 (0.17 μg/kg), X2 (0.12 μg/kg), and X9 (0.14 μg/kg). All other samples showed no detectable ochratoxin-A. Total aflatoxins (B1 + B2 + G1 + G2) were not detected in any sample. The most commonly detected pesticide residue was deltamethrin, found in samples X2 (0.059 mg/kg), X3 (0.050 mg/kg), X4 (0.015 mg/kg), and X6 (0.057 mg/kg). Bifenthrin was detected only in X3 (0.012 mg/kg). Chlorpyrifos ethyl was detected in X3 (0.009 mg/kg) and X6 (0.008 mg/kg). All other samples showed no detectable pesticide residues. All detected pesticide concentrations were below Codex Alimentarius Maximum Residue Limits (MRLs) for wheat: deltamethrin (MRL 0.20 mg/kg), bifenthrin (MRL 0.10 mg/kg), and chlorpyrifos ethyl (MRL 0.01 mg/kg). However, the detection of chlorpyrifos ethyl in samples X3 and X6 is noteworthy, as this compound is banned or severely restricted in many countries.

**Table 3:** Mycotoxin and pesticide residue levels in selected wheat flour samples.

Sample description	Ochratoxin-A (μg/kg)	Total Aflatoxins (μg/kg)	Deltamethrin (mg/kg)	Bifenthrin (mg/kg)	Chlorpyrifos ethyl (mg/kg)
X 1	0.17	ND*	ND	ND	ND
X 2	0.12	ND	0.059	ND	ND
X 3	ND	ND	0.050	0.012	0.009
X 4	ND	ND	0.015	ND	ND
X 5	ND	ND	ND	ND	ND
X 6	ND	ND	0.057	ND	0.008
X 7	ND	ND	ND	ND	ND
X 8	ND	ND	ND	ND	ND
X 9	0.14	ND	ND	ND	ND
X 10	ND	ND	ND	ND	ND
X 11	ND	ND	ND	ND	ND
X 12	<10	<10	ND	<10	ND
X 13	ND	ND	ND	ND	ND
X 14	ND	ND	ND	ND	ND

ND* = Not Detected. LOD: Ochratoxin-A = 0.1 μg/kg; Aflatoxins = 0.5 μg/kg; Deltamethrin = 0.005 mg/kg; Bifenthrin = 0.005 mg/kg; Chlorpyrifos ethyl = 0.003 mg/kg.

### 3.4 Bacterial isolation and phenotypic characterization

A total of 34 bacterial isolates were initially recovered from the 14 flour samples on NA and TSA plates. Of these, 23 morphologically distinct isolates (designated A1–A23) were successfully purified through repeated subculturing and were selected for further characterization. The remaining 11 isolates were not included in this study due to redundancy with already-purified isolates. Complete phenotypic characterization was done on all isolates. Of the 23 isolates, 20 (87.0%) were Gram-positive and 3 (13.0%) were Gram-negative. Cell shapes included cocci (*n* = 19, 82.6%), rods (*n* = 3, 13.0%), and coccobacilli (*n* = 1, 4.3%). All 23 isolates (100%) were catalase positive. Oxidase activity was observed only in *Psychrobacter sanguinis* (A3), *Allohumibacter endophyticus* (A5), and *Delftia lacustris* (A18). The majority of isolates (20/23, 87.0%) exhibited gamma-hemolysis (non-hemolytic) on sheep blood agar. However, three isolates *Bacillus paramycoides* (A2 and A15) and *Peribacillus frigoritolerans* (A9)—demonstrated beta-hemolysis (complete lysis of red blood cells). No alpha-hemolytic isolates were detected.

### 3.5 Molecular identification

**Figure 2:**
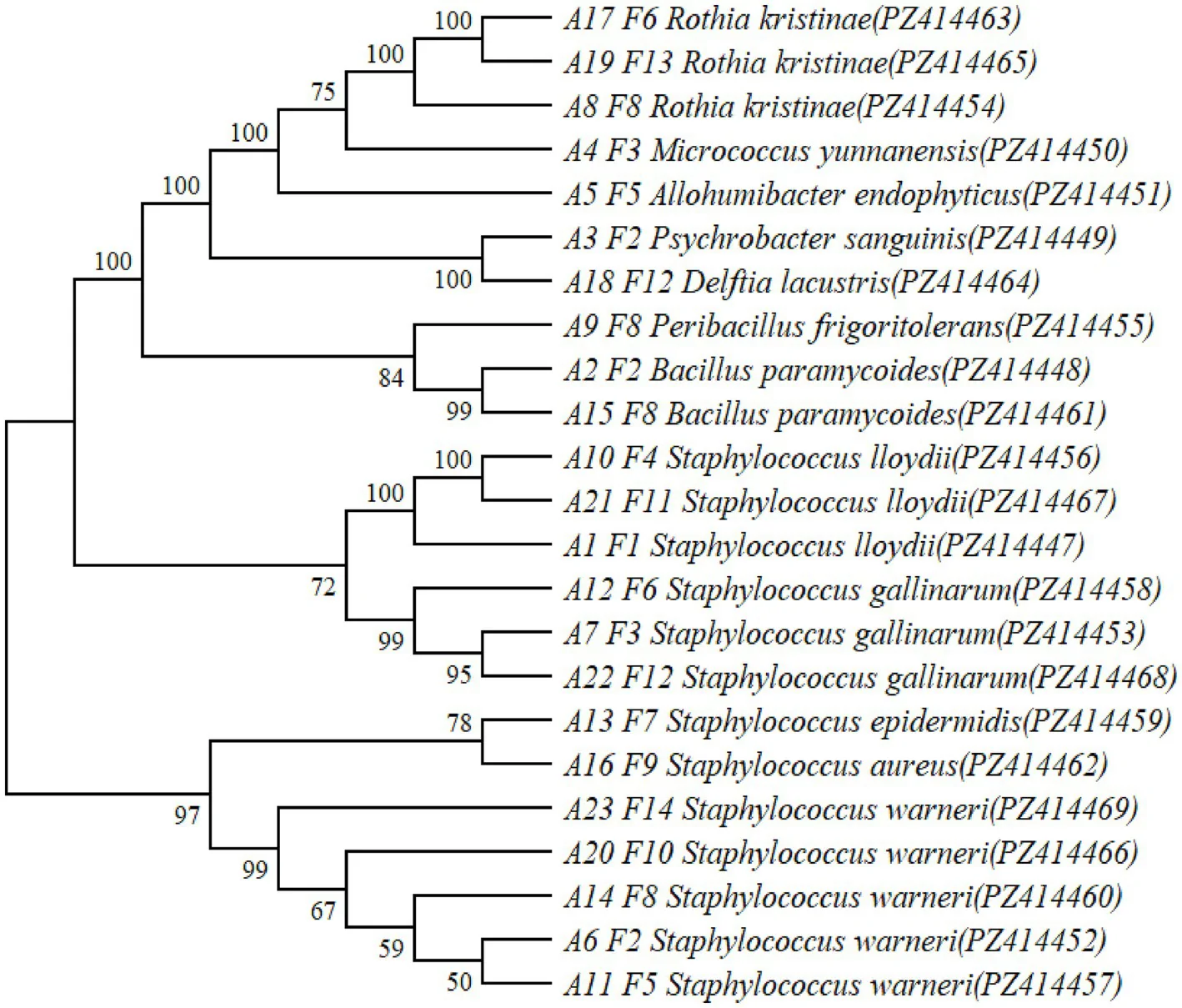
Phylogenetic tree of 16S rRNA gene sequences showing the relationship among bacterial isolates from wheat flour.

The 23 bacterial isolates were identified by 16S rRNA gene Sanger sequencing. Phylogenetic trees were constructed using MEGA12 (Figure 2). Bootstrap values ≥70% were considered well-supported; lower values were interpreted with caution.

**Figure 3:**
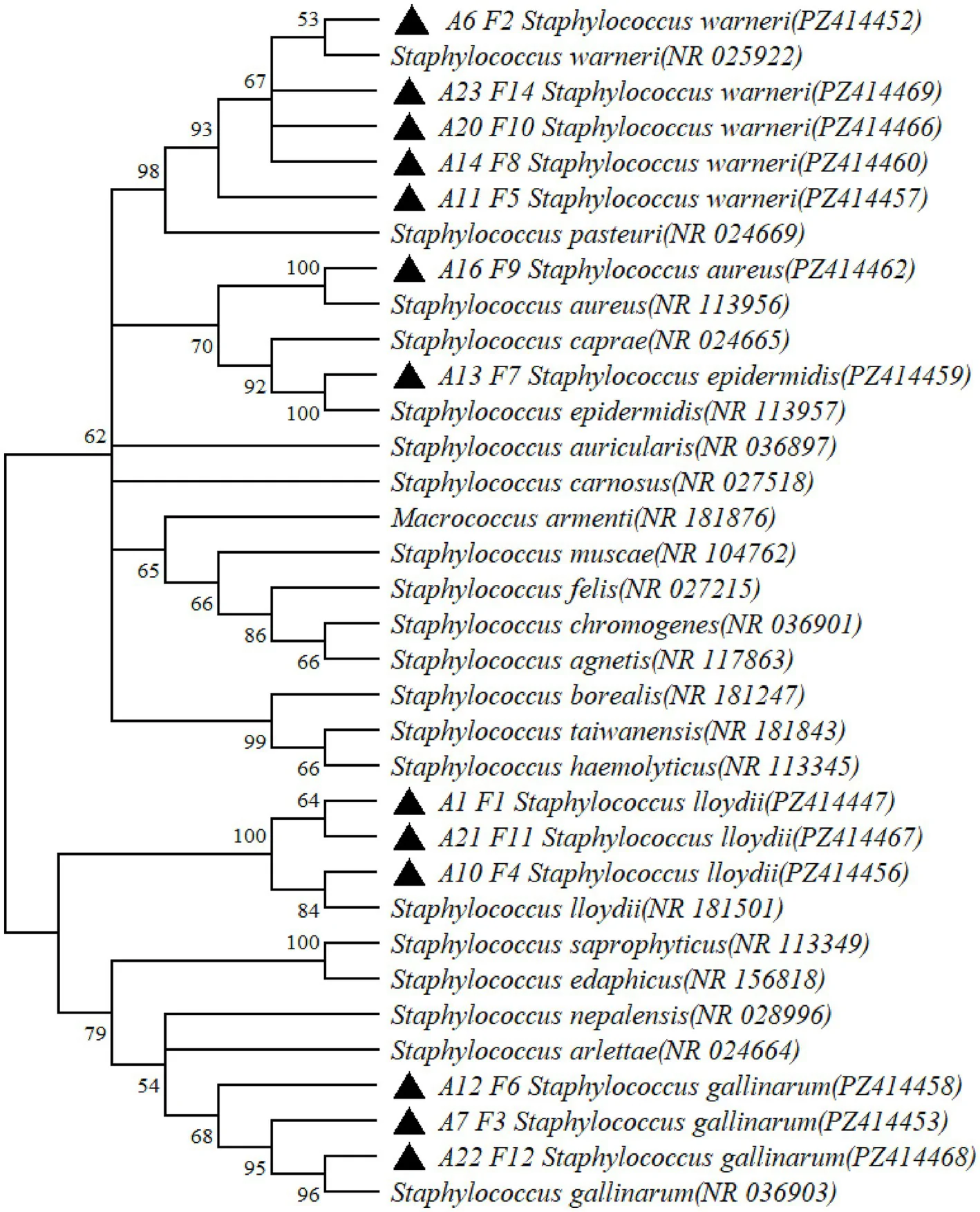
Phylogenetic placement of *Staphylococcus* isolates (*S. warneri*, *Staphylococcus lloydii*, *S. gallinarum*, *S. epidermidis*, *S. aureus*) based on 16S rRNA gene sequencing. Bootstrap values (≥60%) are shown at nodes.

Figure 3 showed that *Staphylococcus* predominated, including *S. warneri* (A6, A11, A14, A20, A23; PZ414452, PZ414457, PZ414460, PZ414466, PZ414469, *Staphylococcus lloydii* A1, A10, A21; PZ414447, PZ414456, PZ414467; 53%–99%), *S. gallinarum* (A7, A12, A22; PZ414453, PZ414458, PZ414468), *S. epidermidis* (A13; PZ414459), and *S. aureus* (A16; PZ414462), bootstrap support ranged from 60% to 100% for most clades.

**Figure 4:**
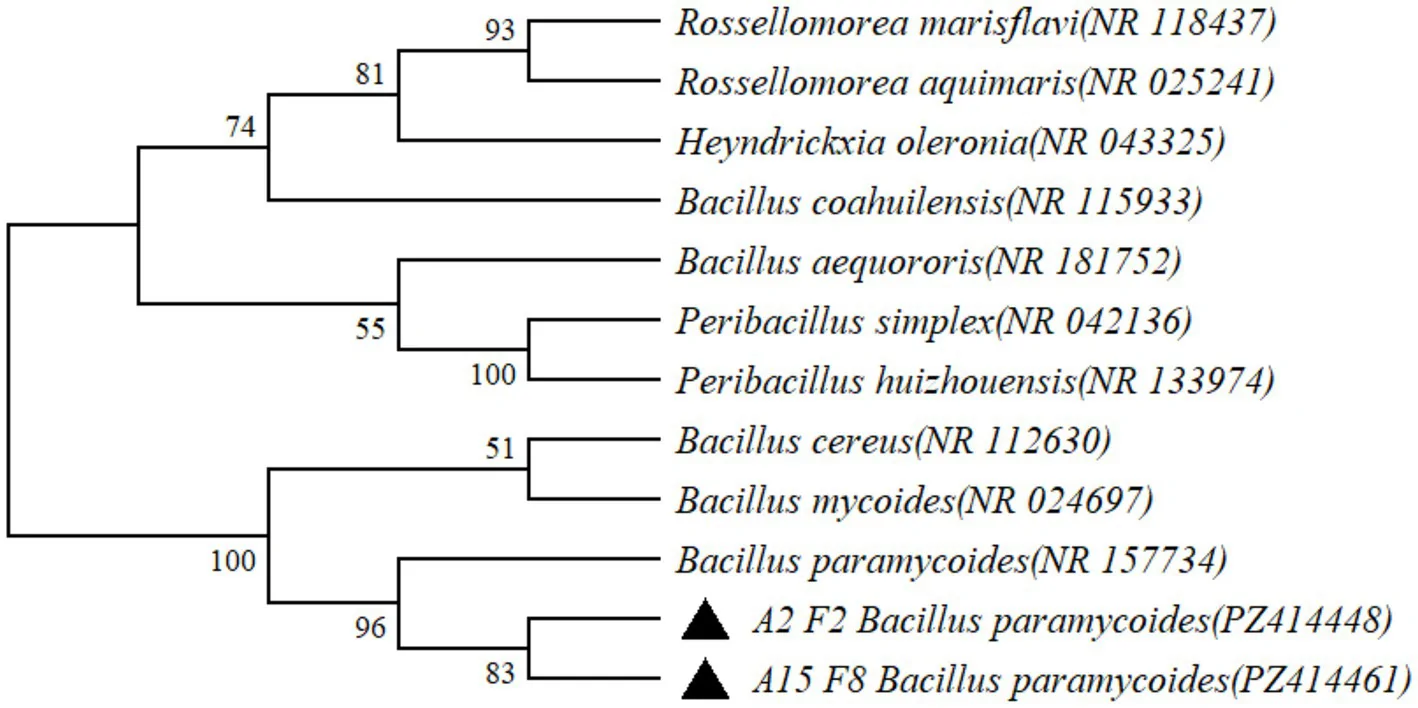
Phylogenetic tree confirming the identification of *Bacillus paramycoides* isolates (A2, A15).

**Figure 5:**
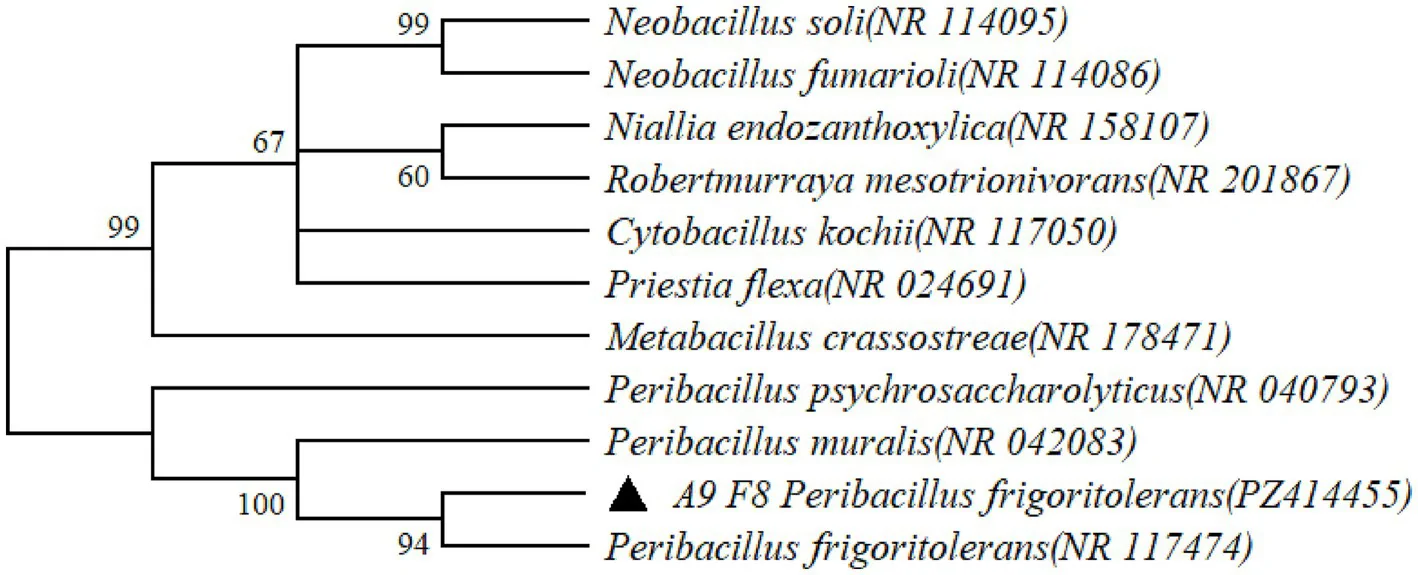
Phylogenetic identification of *Peribacillus frigoritolerans* isolate A9 based on 16S rRNA gene sequence analysis.

**Figure 6:**
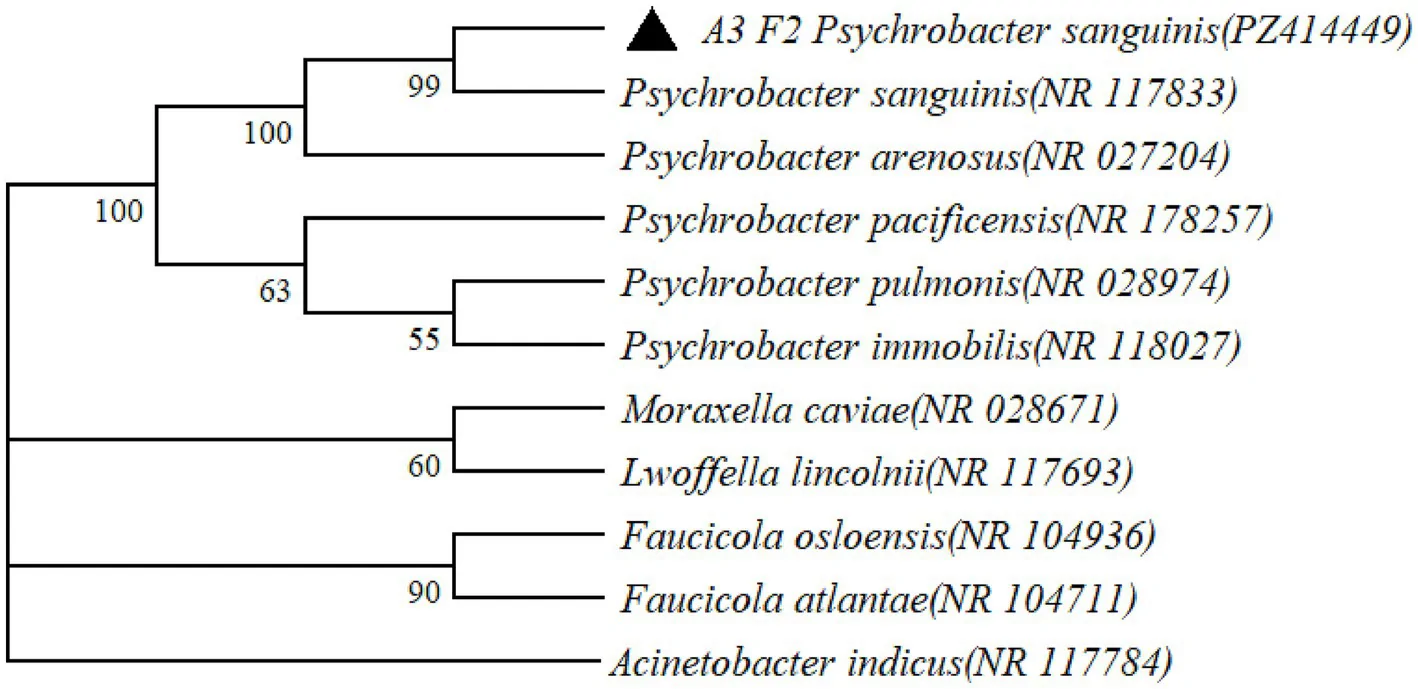
Phylogenetic tree showing the placement of *Psychrobacter sanguinis* isolate A3 with high bootstrap support (96%–100%).

**Figure 7:**
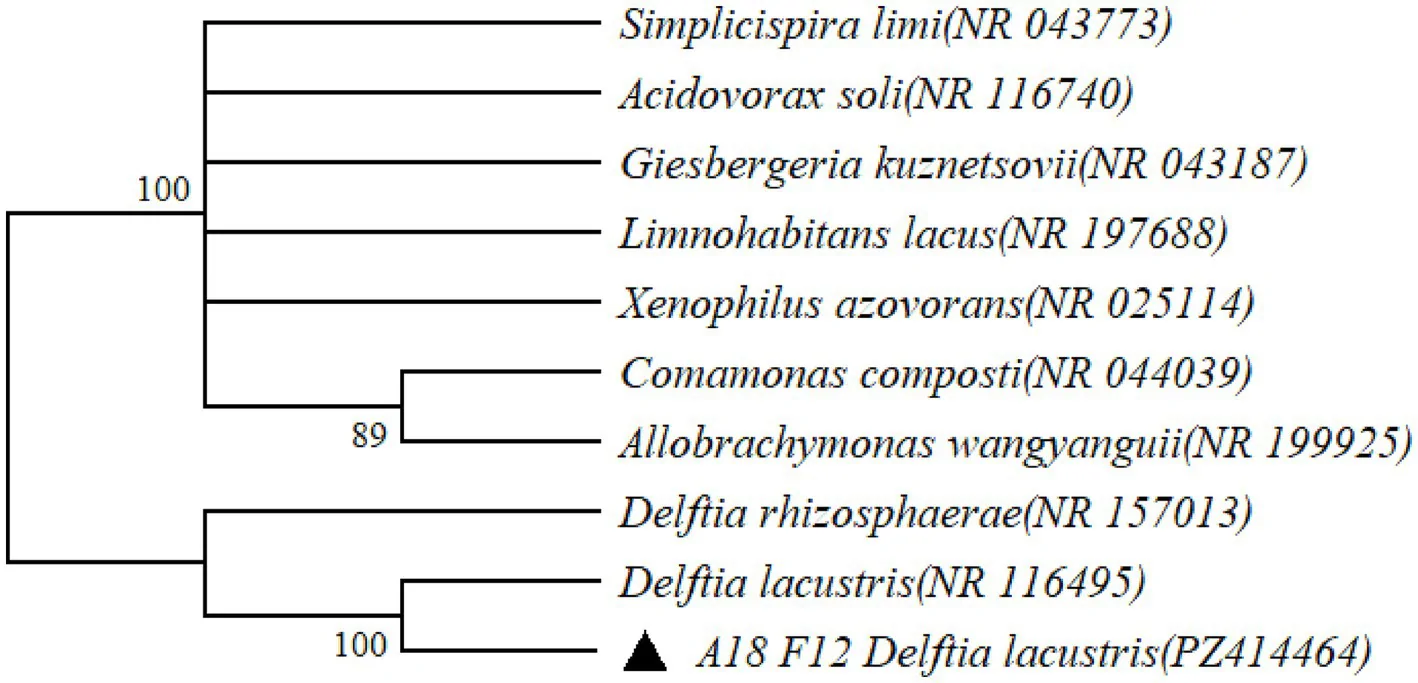
Molecular identification of *Delftia lacustris* isolate A18 by 16S rRNA gene sequencing (bootstrap 94%–95%).

**Figure 8:**
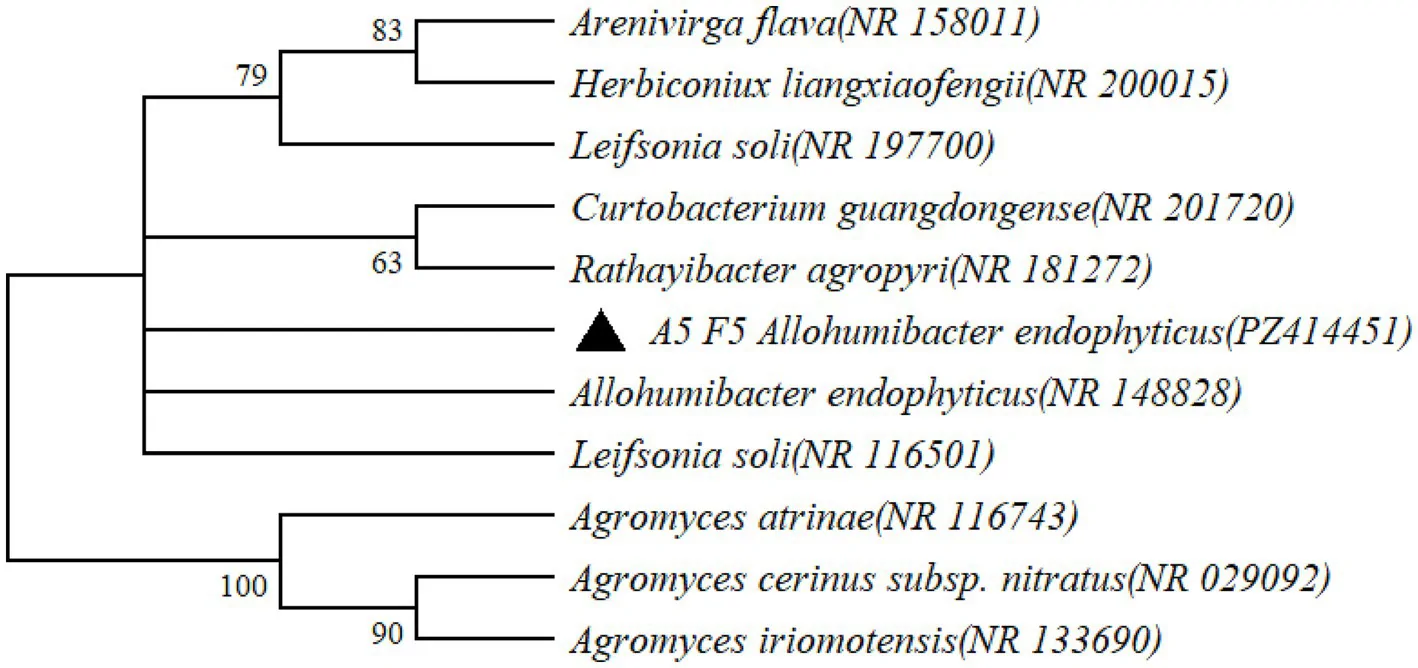
Phylogenetic tree of *Allohumibacter endophyticus* isolate A5.

**Figure 9:**
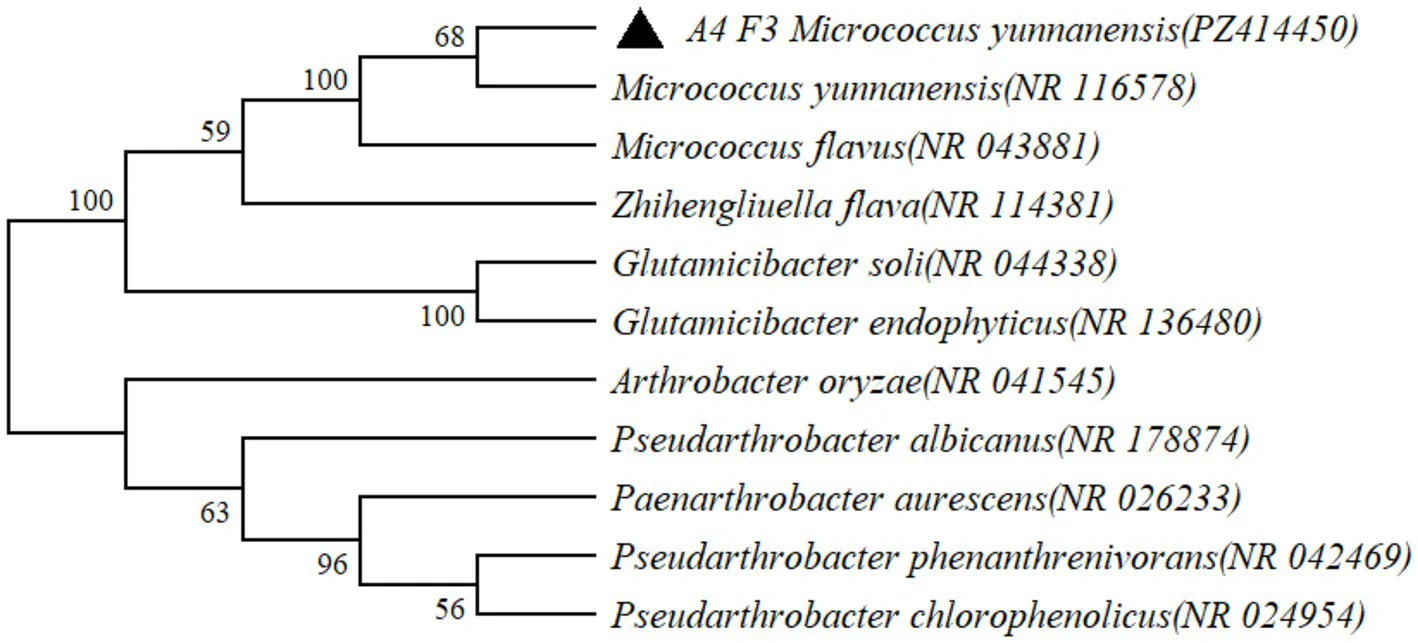
Identification of *Micrococcus yunnanensis* isolate A4 based on 16S rRNA gene phylogeny.

**Figure 10:**
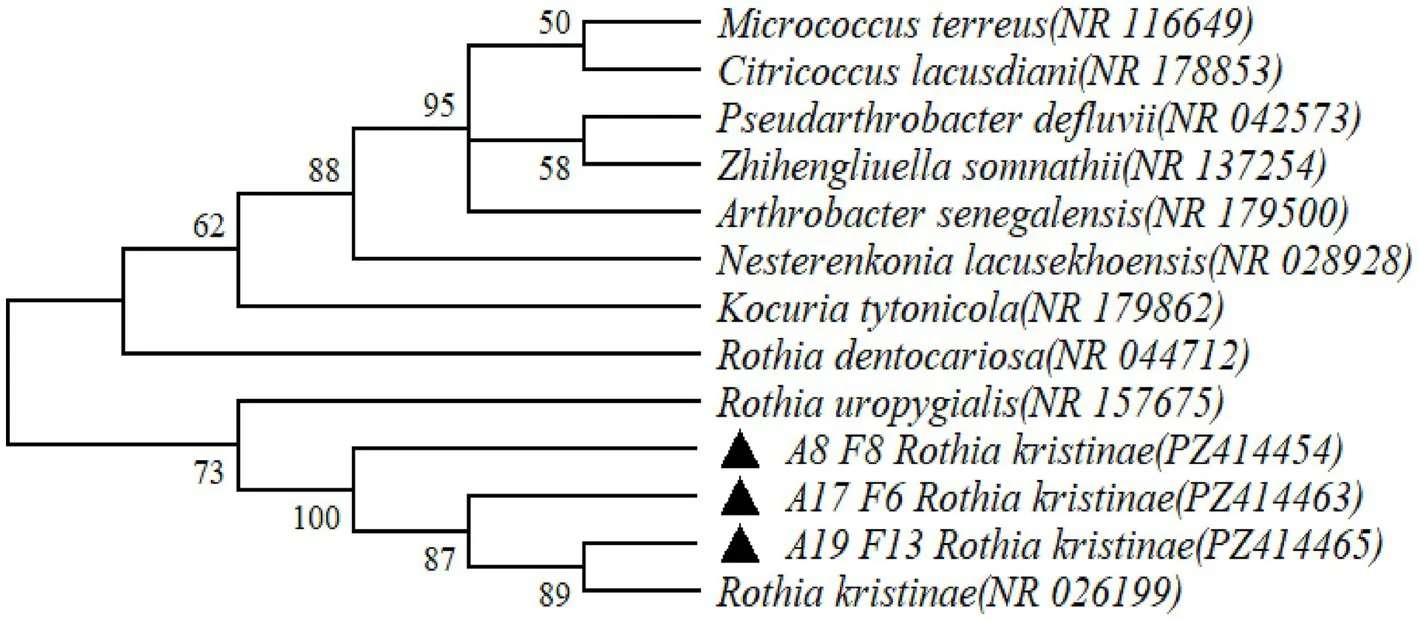
Phylogenetic tree of *Rothia kristinae* isolates (A8–A19) with bootstrap values ranging from 85% to 100%.

Figure 4 confirmed *Bacillus paramycoides* (A2, A15; PZ414448, PZ414461) with 100% bootstrap support. Figure 5 identified *Peribacillus frigoritolerans* (A9; PZ414455). Figure 6 placed *Psychrobacter sanguinis* (A3; PZ414449) with 96%–100% bootstrap support. Figure 7 identified *Delftia lacustris* (A18; PZ414464) with 94%–95% bootstrap support. Figure 8 identified *Allohumibacter endophyticus* (A5; PZ414451) with 100% bootstrap support. Figure 9 identified *Micrococcus yunnanensis* (A4; PZ414450). Figure 10 placed three isolates as *Rothia kristinae* (A8, A17, A19; PZ414454, PZ414463, PZ414465) with 85–100% bootstrap support. All assignments were supported by high bootstrap values, and sequences were deposited under GenBank accessions PZ414447–PZ414469 (release date: May 23, 2026).

## 4 Discussion

This study provides a preliminary baseline assessment of microbiological quality, heavy metal, mycotoxin, and pesticides of commercial wheat flour samples in Jeddah market. Combined with molecular characterization of associated bacterial communities.

### 4.1 Microbiological quality

The absence of *Salmonella* spp. in all 25 g samples and the low levels of *B. cereus*, *E. coli*, and *S. aureus* (<10 CFU/g) indicate that commercial wheat flour in Saudi Arabia complies with international food safety standards for these pathogens. These findings are consistent with studies from other countries. A five-year monitoring project in Türkiye of 984 wheat samples revealed that only 4.77% of samples contained detectable pesticide residues, with most samples meeting safety standards (Yiğitsoy and Sadighfard, 2025). Similarly, a study of mycotoxins in wheat flour produced in Ardabil, Iran, found that all eight mycotoxins analyzed were below acceptable levels (Davari et al., 2025).

The absence of *E. coli* (<10 CFU/g) in all samples is particularly noteworthy, as *E. coli* serves as an indicator of fecal contamination and inadequate sanitation during processing. This observation suggests that milling facilities serving the Saudi market maintain sufficient hygienic standards. However, the detection of elevated yeast and mold counts in sample X8 (830 CFU/g) is concerning and may indicate improper storage conditions or extended shelf life. This sample was identified as a statistical outlier, and its source should be investigated to prevent recurrence. Nevertheless, according to Doddabematti Prakash et al. (2025), contamination of wheat flour is one of the most serious issues in the sphere of food safety, which needs to be monitored and prevented.

### 4.2 Mycotoxin contamination

Analysis of mycotoxins showed that no Total Aflatoxins (B1 + B2 + G1 + G2) were detected in any of the 14 samples, which is a very good result since hepatotoxic, carcinogenic and immunosuppressive effects of aflatoxins are well documented. Then, Ochratoxin-A was detected in three samples (X1: 0.17 μg/kg; X2: 0.12 μg/kg; X9: 0.14 μg/kg), all below the maximum limits set by regulatory institutions (EU: 3.0 μg/kg; China: 5.0 μg/kg) (Cheng et al., 2025). In the same vein, Iranian National Standards Organization has set allowable thresholds that can accommodate such low concentrations (Davari et al., 2025).

The low level of ochratoxin A in this study is in line with local findings. A thorough examination of mycotoxin contamination in major cereals (corn, wheat, and rice) in various regions carried out by Niaz et al. (2025) revealed that Asia had high levels of deoxynivalenol (55.25) and ochratoxin A (47.24). It was also reported that considerable differences by region were found, with the highest levels of contamination in Africa (36.59%), Asia (33.06%), Europe (29.06%), and the Americas (19.20%). Deoxynivalenol demonstrated the greatest average contamination rate in all regions 49.6% (Niaz et al., 2025).

It should be noted that this study did not examine deoxynivalenol (DON), which is the most significant mycotoxin contaminant in wheat flour globally (Cheng et al., 2025). The presence of ochratoxin A in three samples, albeit at low concentrations, warrants follow-up monitoring, as *Aspergillus* and *Penicillium* species can produce ochratoxin A during improper storage. Liu et al. (2025) emphasized that mycotoxin contamination poses a serious threat to grain safety and human health, highlighting the need for routine monitoring across different flour types and seasons.

Liu et al. (2025) pointed out that mycotoxin contamination has become a serious issue that endangers the safety of raw materials in grains and human health because such toxic secondary metabolites generated by fungi may lead to serious health effects and become a huge threat to population health. It was suggested that the study adopt routine observation of the different types of flour in different seasons because there may be dissimilarity in the supply of wheat in different regions or other overseas sources (Liu et al., 2025).

### 4.3 Pesticide residue contamination

The most detected pesticide was deltamethrin, found in samples X2 (0.059 mg/kg), X3 (0.050 mg/kg), X4 (0.015 mg/kg), and X6 (0.057 mg/kg). Chlorpyrifos ethyl was detected in X3 (0.009 mg/kg) and X6 (0.008 mg/kg), while bifenthrin was found only in X3 (0.012 mg/kg). All detected concentrations were below Codex Alimentarius MRLs: deltamethrin (0.20 mg/kg), bifenthrin (0.10 mg/kg), and chlorpyrifos ethyl (0.01 mg/kg).

These findings align with a study by Mishra et al. (2025), who analyzed 96 pesticides and metabolites in 36 wheat samples from Haryana, India, and identified chlorpyrifos, deltamethrin, malathion, and fenvalerate at concentrations below FSSAI maximum residue limits. The Health Risk Index values ranged from 0.001 to 0.129, all below 1, indicating no serious health risk to consumers (Mishra et al., 2025).

However, the detection of chlorpyrifos ethyl is concerning because this compound is banned or severely restricted in many countries. A multi-year monitoring study in Türkiye (2021–2024) on 984 wheat samples revealed detectable pesticide residues in 4.77% of samples, with 2.54% exceeding EU MRLs (Yiğitsoy and Sadighfard, 2025). Chlorpyrifos-ethyl was the most found pesticide and exceeded its MRL in all positive samples. Notably, chlorpyrifos-ethyl was prohibited in Türkiye in 2020, but its continued detection indicates either illegal use or persistence in agricultural soils (Yiğitsoy and Sadighfard, 2025). This observation is directly relevant to the Gulf region, as Türkiye is a major exporter of wheat-based products, ranking first globally in flour exports and second in pasta exports. Between 2021 and 2025, the Rapid Alert System for Food and Feed (RASFF) issued numerous alerts for wheat and wheat-derived products from countries including Ukraine, Jordan, and Slovakia due to unauthorized or excessive chlorpyrifos levels (Yiğitsoy and Sadighfard, 2025).

In November 2025, around 200 new maximum residues of different pesticides were approved by the 56th session of the Codex Alimentarius Committee on Pesticide Residues (CCPR56) and new principles of monitoring the purity and stability of reference materials were finalized. These changes highlight the evolving nature of international pesticide regulation and the need to harmonize national standards to the Codex recommendations (Leprêtre, 2025).

### 4.4 Heavy metal contamination

Statistical analysis revealed that lead levels varied considerably, with a mean of 0.128 ± 0.158 mg/kg. The sample X1 (Brown Flour, 0.566 mg/kg) as a significant outlier (G = 3.42, *p* < 0.05). After exclusion of this outlier, the mean lead concentration was 0.065 ± 0.052 mg/kg, which was significantly below the GSO limit of 0.20 mg/kg (one-sample *t*-test, *t* = 2.50, *df* = 12, *p* = 0.030). Cadmium levels (0.033 ± 0.014 mg/kg) were also significantly below the GSO limit of 0.10 mg/kg (*t* = −18.4, *df* = 13, *p* < 0.001).

The elevated lead level in sample X1 (0.566 mg/kg) exceeds the GSO limit of 0.20 mg/kg and warrants investigation. Possible sources include soil contamination, atmospheric deposition from industrial or traffic sources, use of contaminated irrigation water, or processing equipment abrasion. Mustfa and Ahmed Albarwary (2025) reported detectable lead levels ranging from 0.539 to 1.035 mg/kg in wheat grains grown near industrial areas in Duhok City, Iraq, suggesting that proximity to industrial zones is a significant risk factor.

No statistically significant differences were observed between refined and whole wheat flour groups for any heavy metal (*p* > 0.05 for all comparisons), suggesting that flour type alone does not predict contaminant levels. This contrasts with some studies that have reported higher heavy metal concentrations in whole grain products due to the retention of metal-rich bran layers, and warrants further investigation with a larger sample size.

### 4.5 Bacterial diversity

This 16S rRNA gene sequencing study has generated 23 new sequence accessions for the culturable bacterial community found in the wheat flour of Saudi Arabia that have been deposited in GenBank (PZ414447–PZ414469). A wide variety of bacteria were identified in commercial wheat flour of Saudi Arabia, with the highest prevalence of *Staphylococcus* in which *Staphylococcus warneri*, *Staphylococcus lloydii*, *Staphylococcus gallinarum*, *Staphylococcus epidermidis* and *Staphylococcus aureus* were the most common species.

All the staphylococcal isolates were non-hemolytic. But, a non-hemolytic reaction does not ensure safety. Foodborne staphylococci are often also resistant to antibiotics and able to form biofilms, with *S. epidermidis* and *S. aureus* being strong biofilm formers (Marino et al., 2011). The presence of *S. aureus* (A16), even in low levels (<10 CFU/g), is a cause for concern because this organism has been linked to food poisoning that has occurred as a result of raw consumption of flour in food production (Magallanes Lopez and Simsek, 2021).

More alarmingly, all three Bacillus isolates (*Bacillus paramycoides* A2, A15 and *Peribacillus frigoritolerans* A9) showed beta-hemolysis. *β*-Hemolysis on sheep blood agar indicates the production of pore-forming toxins (hemolysins) that lyse red blood cells, a phenotype associated with the virulence of *B. cereus* and related species. However, β-hemolysis alone does not confirm enterotoxigenicity or clinical pathogenicity. The *B. cereus* group, which includes *B. paramycoides*, is known to produce enterotoxins (*Nhe*, *Hbl*, *CytK*) that can cause diarrheal illness in humans (Jovanovic et al., 2021). While we have not confirmed the presence of these enterotoxin genes, the β-hemolytic phenotype observed in Bacillus isolates from flour samples X1 (Brown Flour) and X9 (Super Superior Brown Flour) suggests that flour could serve as a vehicle for potentially virulent Bacillus strains. This finding underscores the need for routine monitoring of flour for Bacillus species, particularly in products intended for consumption without adequate heat treatment.

Other environmental isolates (*Rothia*, *Psychrobacter*, *Allohumibacter*, *Micrococcus* and *Delftia*) were non-hemolytic which is in line with their low virulence potential as reported by Sulieman et al. (2022). The newly deposited GenBank accessions (PZ414447–PZ414469) will serve as a useful tool in the future for the monitoring of food safety in the Gulf region.

### 4.6 Limitations of the study and future directions

Several limitations should be considered when interpreting these findings. First, the sample size was limited (*n* = 14) and samples were collected only from Jeddah retail markets, providing useful preliminary data but not representing the full diversity of flour products across the Kingdom. Second, the mycotoxin analysis was restricted to aflatoxins and ochratoxin-A; deoxynivalenol (DON), zearalenone, and T-2/HT-2 toxins—among the most prevalent mycotoxin hazards in wheat globally were not analyzed. Third, species-level identification using 16S rRNA gene sequencing has inherent limitations within closely related genera such as *Staphylococcus* and the *Bacillus cereus* group, where interspecies sequence identity can exceed 99%; definitive resolution would require multi-locus sequencing or whole genome sequencing. Fourth, *β*-hemolysis is a preliminary phenotypic marker and does not definitively establish enterotoxigenicity; confirmation requires PCR screening for enterotoxin genes (nhe, hbl, cytK) or cytotoxicity assays. Finally, molecular confirmation of antibiotic resistance genes (*mecA*, *van* genes) was not performed. Future studies should include broader geographic and seasonal coverage, full mycotoxin panels, genotypic virulence and resistance validation, and comprehensive health risk assessment.

## 5 Conclusion

This preliminary baseline assessment of 14 commercial wheat flour samples from the Saudi market demonstrates that most samples complied with international food safety standards for pathogenic bacteria, aflatoxins, and heavy metals. However, several findings warrant attention: elevated lead in one sample (X1, 0.566 mg/kg) exceeding GSO limits; chlorpyrifos ethyl detected in two samples; beta-hemolytic *Bacillus* isolates suggesting potential virulence markers; and elevated yeast/mold counts in one sample (X8, 830 CFU/g). 16S rRNA sequencing revealed a diverse bacterial community dominated by *Staphylococcus* (56.5%), with 23 new GenBank accessions deposited. We recommend routine monitoring of imported flour for banned pesticides, investigation of lead contamination sources, larger multi-regional studies with full mycotoxin panels, genotypic virulence screening, and implementation of regular monitoring aligned with Codex and GCC regulations.

## Data Availability

The original contributions presented in the study are included in the article/supplementary material, further inquiries can be directed to the corresponding authors.
